# Nutritional Strategies for Enhancing Performance and Training Adaptation in Weightlifters

**DOI:** 10.3390/ijms26010240

**Published:** 2024-12-30

**Authors:** Dong-Joo Hwang, Hong-Jun Yang

**Affiliations:** 1 Sport Science Institute, Korea National Sport University, Seoul 05541, Republic of Korea; dongzoo87@knsu.ac.kr; 2 Institute of Health & Environment, Seoul National University, Seoul 08826, Republic of Korea

**Keywords:** weightlifting, training adaptation, nutrition, recovery, supplements

## Abstract

Weightlifting demands explosive power and neuromuscular coordination in brief, repeated intervals. These physiological demands underscore the critical role of nutrition, not only in optimizing performance during competitions but also in supporting athletes’ rigorous training adaptations and ensuring effective recovery between sessions. As weightlifters strive to enhance their performance, well-structured nutritional strategies are indispensable. In this comprehensive review, we explored how weightlifters can optimize their performance through targeted nutritional strategies, including carbohydrate intake for glycogen replenishment and proteins for muscle growth and recovery. Additionally, the roles of key supplements, such as creatine, beta-alanine, and branch-chained amino acids in enhancing strength, delaying fatigue, and supporting muscle repair were discussed. A comprehensive literature review was conducted using PubMed, Google Scholar, and Web of Science to gather studies on nutritional strategies for weightlifting performance and training adaptation. The review focused on English-language articles relevant to weightlifters, including studies on powerlifting, while excluding those involving non-human subjects. Weightlifting requires explosive power, and proper nutrition is vital for performance and recovery, emphasizing the role of carbohydrate, protein, and fat intake. Nutrient timing and personalized strategies, informed by genetic and metabolomic analyses, enhance recovery and performance, while supplements like creatine, caffeine, and beta-alanine can significantly improve results when used correctly. Sustainable nutritional strategies are essential for enhancing weightlifter performance, emphasizing a balanced approach over extreme diets or excessive supplements. Further research is needed to refine these strategies based on individual athlete characteristics, ensuring consistent top-level performance throughout competitive seasons.

## 1. Introduction

Weightlifting, one of the most historic sports in the modern Olympic Games, requires athletes to generate maximum strength in an extremely short timeframe, demonstrating both explosive power and neuromuscular coordination [1]. As an event open to both men and women, countless elite athletes worldwide prepare their physical and technical training to achieve the best results at this highly competitive stage. Olympic-level weightlifting requires immense strength, explosive power, and excellent neuromuscular coordination, all of which depend heavily on rigorous training and proper nutritional management [2,3].

Given that Olympic weightlifters must utilize their muscles to the fullest extent within a brief timeframe, customized nutritional strategies are essential to maximize physical performance. Recent research suggests that nutrition goes beyond physical maintenance and is now recognized as a direct factor influencing performance [4]. Various physical functions, such as strength development, muscle recovery, fatigue recovery, and metabolic efficiency, are determined by nutritional status, and in high-stakes competitions, such as the Olympics, nutritional strategies can be key variables in determining success or failure [5,6]. The short bursts of power required for weightlifting predominantly rely on the adenosine triphosphate–phosphocreatine (ATP-PC) energy system, which provides rapid energy replenishment for maximum effort. Consequently, proper carbohydrate loading before competition and consistent protein intake for muscle repair are fundamental strategies that contribute to sustained performance, particularly during events requiring multiple attempts over a short timeframe [7,8]. Additionally, post-exercise nutrition plays a pivotal role in recovery, as consuming adequate carbohydrates and proteins shortly after training enhances muscle glycogen replenishment and promotes muscle protein synthesis (MPS), which is crucial for minimizing muscle damage and accelerating recovery [9,10,11]. Studies have emphasized that ingesting high-quality proteins, such as whey, immediately after exercise can significantly improve muscle recovery and promote hypertrophy [12,13].

Strategic use of dietary supplements has gained prominence in the weightlifting world. Creatine supplementation is one of the most extensively researched ergogenic aids recognized for its ability to increase intramuscular phosphocreatine stores, which enhance power output during short-duration, high-intensity activities [14,15]. Similarly, beta-alanine is widely used to buffer muscle acidity during repeated high-intensity efforts, thereby delaying the onset of muscle fatigue [16,17]. Although the benefits of supplements are well-supported, issues, such as long-term safety and variability in individual responses, must be carefully considered [18,19]. Hydration and micronutrient balance are critical for optimal performance and recovery, particularly under challenging environmental conditions. Electrolyte imbalance and dehydration can negatively affect neuromuscular function, making individualized hydration strategies essential [20,21]. Moreover, micronutrients, such as magnesium and vitamin D, support neuromuscular health and mitigate stress responses, which are critical for maintaining both physical and psychological performance under the pressure of elite competition [21].

The future of sports nutrition lies in personalized nutrition approaches. Advances in genomics and metabolomics allow tailored nutritional interventions to address the unique metabolic needs of each athlete. This shift towards individualized care and optimizing nutrients according to genetic profiles and training demands represents a key area in sports science that can enhance both training adaptations and competitive performance [22,23]. Personalized nutrition offers a promising avenue for improving immediate performance outcomes and supporting the long-term health and sustainability of elite athletes.

This review summarizes current research on nutritional strategies specifically tailored to weightlifting. By exploring the roles of key macronutrients, supplements, nutrient timing, and emerging trends in personalized nutrition, this study provides practical guidelines for optimizing the training and competitive performance of elite weightlifters.

## 2. Methods

### 2.1. Literature Review

A comprehensive literature review was conducted to identify relevant studies related to the research topic (nutritional strategies for weightlifting performance and training adaptation). The PubMed, Google Scholar, and Web of Science databases were systematically searched for the research findings cited in this review. These databases were selected because of their broad and multidisciplinary coverage, which ensured access to a wide range of peer-reviewed articles (literature and systemic), clinical trials, and conference proceedings. PubMed was selected for its focus on the biomedical and life science literature, Web of Science was selected for its high-impact, multidisciplinary content, and Google Scholar was selected for its accessibility to a broad spectrum of academic sources. Additional literature was obtained by screening the reference lists of the included articles. The predefined inclusion criteria for all articles were as follows: (i) referred articles available in English and (ii) the presence of terms related to weightlifting’, ‘weightlifters’, or ‘athlete’, given the limited research specifically focused on weightlifters. Studies on powerlifting and other high-intensity exercises were included to reflect the similar movement patterns and physiological demands experienced by weightlifters. Studies conducted on animals or non-human subjects were excluded.

### 2.2. Search Terms

The search strategy incorporated keywords and subject-specific terms relevant to the research objectives, ensuring comprehensive retrieval of studies from the selected databases. The key search terms included ‘weightlifting’, ‘weightlifter(s)’, ‘athletic performance’, ‘intense exercise (or high-intensity)’, ‘training adaptation’, ‘nutrition’, ‘nutrient(s)’, ‘nutritional strategy’, ‘nutrient timing’, ‘supplement(s)’, ‘personalized nutrition’, and ‘branched-chain amino acids’. Boolean operators “AND” and “OR” were used to combine these terms appropriately.

## 3. Advances in Exercise Nutrition: Precision Approaches for Weightlifting Athletes

### 3.1. Recent Trends in Exercise Nutritional Research

In recent years, interest in personalized nutrition has grown significantly in sports science and nutrition. Tailored nutritional strategies that consider an athlete’s physiological characteristics, genetic profile, and metabolic state are now recognized as critical factors for improving performance, particularly in sports that require explosive power, such as weightlifting [23,24]. Traditional ’generalized’ nutritional approaches fail to sufficiently account for individual differences, and recent research suggests the need for a more personalized approach based on the specific demands of each sport and the genetic and physiological needs of each individual [22].

Advanced technologies, such as next-generation sequencing (NGS), have driven the progress of genomic research, opening new possibilities for designing nutritional strategies tailored to the genetic traits of each weightlifter [25,26,27]. By understanding how specific genes influence protein metabolism, carbohydrate storage, and fat metabolism, it is possible to propose optimal nutrient ratios and timings based on the genetic characteristics of each athlete. For instance, as certain genetic variants significantly affect carbohydrate metabolism, personalized carbohydrate intake strategies that consider these variations can greatly contribute to performance enhancement [25].

Additionally, rapid advancements in metabolomics research have provided tools for precisely analyzing the metabolic changes that occur during and after exercise [28,29]. Metabolomics allows the scientific assessment of an athlete’s metabolic state, including muscle recovery, fatigue accumulation, and nutrient needs, which are critical for establishing optimal nutritional management strategies [29]. This study enables data-driven precision nutrition and offers methodologies to provide personalized nutrition to athletes based on their training intensity and recovery processes. For example, post-exercise metabolite analysis can help in the assessment of muscle damage or glycogen depletion and, based on these insights, can aid in the design of nutritional strategies to optimize recovery [30,31].

Furthermore, these recent studies go beyond simply recommending nutrient intake. They consider intricate details, such as nutrient interactions, timing of intake, and changes in nutritional requirements before and after exercise. This comprehensive approach allows for the development of sophisticated nutritional strategies that maximize performance and promote the rapid recovery of weightlifters, offering a data-driven nutritional management strategy [5]. This is evidence that modern sports nutrition is becoming increasingly precise, and such an approach is essential in high-intensity sports, such as weightlifting [31].

### 3.2. Weightlifting and Energy Metabolism

Weightlifting is a power-driven sport that primarily relies on anaerobic metabolism to supply energy and demands explosive power over a short period. Given these characteristics, it is essential to optimize muscle glycogen storage [9,32]. Glycogen is stored in the muscles and liver, predominantly through carbohydrate intake, and as it is rapidly consumed during training, proper carbohydrate intake is crucial to prevent glycogen depletion and maintain performance. Studies have shown that consuming carbohydrates and proteins after training promotes muscle glycogen re-synthesis and accelerates muscle recovery [9].

During high-intensity exercises, such as weightlifting, lactate quickly accumulates, causing muscle fatigue. This accumulation limits the duration of exercise and delays recovery. Therefore, strategies to reduce muscle fatigue and promote lactate clearance are important. Increasing the lactate threshold through proper nutrition and training strategies helps manage fatigue [33,34]. Additionally, the quick replenishment of glycogen after training accelerates fatigue recovery and contributes to performance optimization in the next training session [9,10,11].

Proteins are essential for muscle development and the recovery of damaged muscles. As weightlifting causes muscle damage, protein intake is necessary for recovery [4]. In particular, the consumption of fast-digesting proteins (e.g., whey protein) immediately after training promotes MPS, which facilitates muscle recovery and growth [35]. Research has suggested that the consumption of 20–30 g of protein after training is optimal for muscle recovery [36].

In conclusion, weightlifters must carefully manage their intake and timing of carbohydrates and protein to effectively supply the energy required for high-intensity exercise. This optimizes muscle glycogen storage and promotes muscle recovery, contributing to performance improvement.

## 4. Major Nutrients

### 4.1. Carbohydrates

Carbohydrates are the primary energy source for weightlifters. Carbohydrates are stored as glycogen in the muscles and liver and rapidly supply energy during high-intensity training. Glycogen is the most important energy source during high-intensity anaerobic exercise, and glycogen depletion in muscles is one of the main factors limiting exercise performance [37]. Typically, weightlifters who perform high-intensity exercise should aim for approximately 55–60% (3–5 g/kg body weight) of their daily caloric intake from carbohydrates to optimize glycogen storage [4,38].

In particular, consuming carbohydrates before training increases muscle glycogen and stores and delays fatigue during training, whereas post-training carbohydrate intake rapidly replenishes depleted glycogen, reduces muscle damage, and enhances recovery speed [9]. Research has shown that consuming high-glycemic carbohydrates immediately after training accelerates glycogen resynthesis and glycogen recovery within 24 h post-training [39,40].

The digestion rate should also be considered when consuming carbohydrates. Fast-digesting, high-glycemic foods (e.g., fruits and sports drinks) are effective in rapidly replenishing muscle glycogen immediately after training. In contrast, slow-digesting complex carbohydrates (e.g., whole grains and high-fiber foods) are advantageous for providing sustained energy before training [41].

### 4.2. Protein

Protein is an essential nutrient for muscle recovery and growth, and adequate protein intake is crucial for optimizing strength development in weightlifters [4]. Studies have suggested that weightlifters should consume 1.6–2.2 g of protein per kilogram of body weight for optimal muscle synthesis and recovery [36,42]. In particular, protein consumption immediately after training maximizes MPS, facilitating rapid recovery from muscle damage [4,43].

Whey protein is considered an ideal post-training supplement because of its rapid absorption. Research has indicated that consuming 20–30 g of whey protein after training promotes muscle synthesis and positively affects strength development [44]. Additionally, the intake of branched-chain amino acids (BCAAs) is important. BCAAs consist of leucine, isoleucine, and valine, which are highly effective in promoting muscle recovery and reducing fatigue. BCAAs are particularly effective in aiding muscle damage recovery and reducing fatigue because they are rapidly absorbed after training to accelerate muscle recovery [45,46,47,48,49].

Furthermore, recent research has suggested that there may not be an upper limit to the anabolic response to protein ingestion during recovery from exercise. Trommelen et al. (2023) found that the body’s anabolic response to protein ingestion, both in terms of magnitude and duration, may continue to increase with higher protein doses, especially during the recovery period following exercise [50]. This finding challenges previous assumptions regarding protein intake caps and highlights the importance of continuous protein consumption during extended recovery periods for optimal muscle protein synthesis [50,51]. Total daily protein intake was the most important factor.

### 4.3. Fat

Although fat plays a less significant role in high-intensity exercises, such as weightlifting, it is still important as a long-term energy source. Fat plays a key role in energy storage and hormone production and is essential for providing the energy needed for recovery after training [4,36,38]. Unsaturated fatty acids reduce inflammation and promote muscle recovery. For example, omega-3 fatty acids exhibit anti-inflammatory properties, promote muscle recovery after exercise, and support nervous system function [52,53].

It is recommended that fat intake should account for 30–40% of the total caloric intake for weightlifters, providing the necessary energy for body maintenance and recovery [36,54]. However, consuming large amounts of fat immediately before or after training is not ideal because of the slow digestion rate. It is preferable to focus on consuming unsaturated fats instead of saturated fats as this helps reduce long-term inflammation in the body and contributes to overall health [55].

## 5. Nutrient Timing Strategies

Nutrient timing plays a crucial role in optimizing the performance of weightlifters. By appropriately supplying nutrients before, during, and after training, athletes can maximize their strength development, recovery, and fatigue reduction [4,49,56,57]. According to the traditional sports nutrition theory, the timing and quantity of nutrient intake have a significant impact on athletic performance, and appropriate timing can enhance muscle recovery and protein synthesis [49]. Timed intake of carbohydrates and proteins plays a key role in glycogen recovery and muscle damage repair [4,57].

### 5.1. Pretraining

Pretraining nutrient intake focuses on optimizing muscle glycogen stores and ensuring sufficient energy for high-intensity training [4,58,59]. Research suggests that consuming a high-carbohydrate meal 2–3 h before training is ideal, with easily digestible and fast-absorbing foods preferable [4,60]. For example, fluid-, gel-, and bar-type foods, as well as whole grains and fruits (e.g., smoothies), can help stabilize blood sugar levels and glycogen stores before training [61].

If glycogen stores are not optimized, fatigue may occur quickly during training, thereby reducing performance capacity. Storey and Smith (2012) emphasized the necessity of adequate carbohydrate intake before training [36]. Additionally, proper hydration before training is critical, and consumption of electrolyte drinks can help maintain electrolyte balance and prevent fluid loss during training [62,63].

### 5.2. During Training

Because weightlifting requires explosive power over a short period of time, additional nutrient supplementation during training is typically unnecessary. However, if the training lasts for more than an hour or is of very high intensity, consuming quickly absorbed carbohydrates may be beneficial [4,63]. Sports drinks or carbohydrate gels can help prevent energy depletion while maintaining performance levels [4,63,64].

Carbohydrate supplementation delays muscle glycogen depletion and reduces fatigue during training [4]. Additionally, replenishing fluids and electrolytes during training helps prevent dehydration due to sweating and can contribute to the prevention of muscle cramps and injuries [62,63].

### 5.3. Post-Training

Post-training nutrient intake is crucial for muscle recovery and glycogen replenishment, with the ideal time for nutrient intake being within 30 min after training. This period is referred to as the “anabolic window of opportunity”, during which muscle protein synthesis and glycogen recovery are most active [9,45,65]. However, the ideal timing for optimal MPS and prevention of loss of muscle mass, along with protein intake, depends on individual tolerance, as it may diminish over time after activity [45,66]. Additionally, while the concept of an ‘anabolic window of opportunity’ suggests a limited timeframe post-exercise for optimal protein intake, numerous studies have challenged this idea, arguing that the window may be more flexible (or not as narrow) than previously proposed, with MPS occurring effectively over a longer period (at least 24 h after a training bout) [56,65,67,68].

Studies suggest that the co-ingestion of carbohydrates and proteins is the most efficient strategy for promoting muscle recovery and glycogen replenishment [9,57,69]. While some studies have challenged the necessity or universal applicability of this approach, arguing that factors, such as the timing of pre-workout meals and individual metabolic responses, may play a critical role, it is still widely accepted as an effective strategy for enhancing recovery after intense training. Foods such as protein shakes paired with bananas or fruit smoothies provide this ideal ratio and, owing to their fast absorption, help accelerate muscle recovery. Whey protein is an effective post-training supplement providing rapidly absorbed proteins that facilitates muscle repair and promotes the formation of new muscle tissues [69,70].

## 6. Practical Nutrient Timing Strategies

Nutrient timing before, during, and after training is critical for performance and recovery, and nutrient intake must be adjusted appropriately at each stage. Below are the specific strategies for nutrient timing before, during, and after training (Table 1):

Pretraining: preparing energy and glycogen storage:

○ Timing: 2–3 h before training;
○ Recommended nutrient ratio: high carbohydrate (~1–4 g/kg body weight), moderate protein (0.15–0.25 g/kg body weight), and low fat [4,45,58];
○ Recommended foods: whole-grain bread or pasta, oatmeal, fruits, such as banana, low-fat yogurt, and smoothies;
○ Example: a bowl of oatmeal with a banana or a fruit smoothie with added nuts or low-fat milk; carbohydrates are essential for glycogen storage during training, protein minimizes muscle damage, and a small amount of fat ensures sustained energy.

During training: energy replenishment and hydration management:

○ Timing: if the training session lasts more than 45 min to 1 h, the athlete will need to consume nutrients at 15–20 min intervals during the session. However, for sessions lasting 45 min or less, carbohydrate replenishment is not necessary [4];
○ Recommended nutrient ratio: fast-absorbing carbohydrates (30–60 g/h) + fluids (500–1000 mL/h) [63];
○ Recommended supplements: sports drinks (containing 6–8% carbohydrates), carbohydrate gels, and electrolyte drinks [63];
○ Example: 500 mL of a sports drink and 1–2 carbohydrate gels (adjusted based on training duration); fluid replenishment is essential to prevent weight loss exceeding 2% during training, and electrolytes (particularly sodium) must be replaced to prevent dehydration [64]. Carbohydrate intake during training delays glycogen depletion and helps maintain exercise performance [4,63].

Post-training: recovery and replenishment:

○ Timing: within 30 min of training, with an additional meal within 2 h;
○ Recommended nutrient ratio: a carbohydrate–protein ratio of 3:1 or 4:1 (1.0–1.2 g/kg body weight carbohydrates + 0.2–0.4 g/kg body weight of protein) [57,69];
○ Recommended foods: whey protein shakes, fruits (bananas and berries), carbohydrate drinks, smoothies, and whole-grain cereal with milk;
○ Example: whey protein is shaken with a banana or a carbohydrate drink with fruit [45,69]. Carbohydrate intake rapidly replenishes glycogen, whereas protein intake promotes muscle synthesis and accelerates the recovery of damaged muscles [68]. Adequate fluid and electrolyte replenishment also play critical roles in preventing muscle cramps and aiding overall fatigue recovery [63].

## 7. Supplement Use Strategies

Supplements play an important role in helping athletes reduce fatigue during training, promote recovery after exercise, and improve performance [71,72,73]. Below are some key supplements that have been reported to be effective for weightlifters and strategies for their use (Table 2).

### 7.1. Creatine

Creatine is widely used in sports that require explosive power, such as weightlifting, as it enhances strength and power. Creatine plays a key role in the adenosine triphosphate–creatine phosphate (ATP-PC) system by promoting ATP synthesis in muscles to provide rapid bursts of energy [14]. Studies have shown that athletes who take creatine supplements can sustain high-intensity training and recover from fatigue more quickly [2,14,15,74,75].

Usage strategy: regular intake of 3–5 g per day (or approximately 0.3 g/kg body weight) is typically effective, and it can be consumed either before or after exercise. However, a rapid creatine-loading strategy, whereby 20 g per day is taken for 5–7 d, followed by a maintenance phase of 3–5 g per day, has also been shown to effectively enhance performance [75,76]. Related studies have suggested that the loading phase can rapidly saturate muscle creatine stores, leading to an improved high-intensity performance in athletes. Furthermore, long-term supplementation has been associated with enhanced recovery and increased lean body mass, supporting its application in short-term competition preparations and prolonged training [15,75,76,77].

### 7.2. BCAAs

BCAAs, including leucine, isoleucine, and valine, are well known for their roles in promoting muscle recovery and reducing exercise-induced fatigue [44,78]. Studies have highlighted that BCAAs, particularly leucine, are essential for stimulating muscle protein synthesis, repairing damaged muscle tissue, and reducing post-exercise soreness [48,79]. BCAAs are rapidly absorbed after training, enhancing the recovery process, and supporting muscle adaptation [47].

In parallel, BCAAs, particularly leucine, play a crucial role in muscle recovery by activating the mTORC1 signaling pathway, a key regulator of muscle protein synthesis [80]. The activation of mTORC1 leads to the phosphorylation of downstream targets such as S6K1, promoting translational initiation and facilitating muscle recovery and growth. This mechanism ensures that damaged muscle fibers are efficiently repaired and adapted following high-intensity exercise. Together, these processes highlight the essential role of BCAAs in supporting muscle recovery and optimizing performance, particularly in athletes engaged in strength and power-focused sports like weightlifting.

Usage strategy: BCAAs can be consumed before, during, or after exercise, with an effective dosage typically ranging from 5 to 10 g per day, depending on individual needs. The consumption of BCAAs immediately after training can help accelerate muscle repair and mitigate fatigue, particularly in resistance and endurance athletes [48,81].

### 7.3. Beta-Alanine

Beta-alanine increases carnosine levels in skeletal muscles, which plays a key role in reducing the accumulation of hydrogen ions (H^+^) during high-intensity exercise. This accumulation occurs due to anaerobic glycolysis, where lactic acid dissociates into lactate and H^+^, causing a drop in muscle pH (acidosis) and leading to fatigue [82]. By acting as an intracellular pH buffer, elevated carnosine concentrations mitigate this acidification, thereby delaying the onset of acidosis-induced fatigue.

Trexler et al. (2015) demonstrated that beta-alanine is the rate-limiting precursor for carnosine synthesis, and its chronic supplementation significantly increases carnosine content, particularly in type II (fast-twitch) muscle fibers [83,84]. These fibers are predominantly recruited during short, explosive efforts characteristic of weightlifting. Furthermore, studies have shown that beta-alanine supplementation enhances endurance and performance in repetitive high-intensity exercises [16,17,83,84], which are integral to weightlifting. The increase in muscle carnosine levels provides a critical advantage for weightlifters by sustaining force production, delaying neuromuscular fatigue, and maintaining performance during repeated maximal contractions [85,86]. This buffering mechanism is particularly relevant in weightlifting, where short, repeated bursts of effort predominate, underscoring the importance of beta-alanine as an effective ergogenic aid.

Usage strategy: beta-alanine is typically consumed at a dose of 3–6 g per day for 4–8 weeks, gradually increasing the muscle carnosine levels [17,84]. This increase in carnosine levels helps manage long-term fatigue and enhances performance during repeated high-intensity training. Some users may experience paraesthesia (i.e., a tingling sensation) when consuming beta-alanine, which can be attenuated by splitting the dose. For example, administering 4 g/day in two 2 g doses can minimize side effects while still providing the desired benefits [84].

### 7.4. Caffeine

Caffeine is a well-known supplement that reduces fatigue, enhances focus, and improves exercise performance. It is particularly effective in sports, such as weightlifting, which require short bursts of high-intensity effort [83,87,88,89]. It stimulates the central nervous system, reduces the perception of fatigue during exercise, and improves overall efficiency [87]. Goldstein et al. (2010) reported that caffeine is effective for both strength development and endurance exercise, enhancing performance across a wide range of physical activities [87,90].

Usage strategy: caffeine is most effective when consumed 30–60 min before training at a dose of 3–6 mg per kilogram of body weight [88,91,92]. For example, a 70 kg athlete can consume 210–420 mg of caffeine. As caffeine acts quickly, consuming it immediately before training can enhance focus and help maintain training intensity [93]. However, the athletes’ sensitivity to caffeine should be considered because it can affect sleep patterns and digestion. To avoid reduced effectiveness with long-term use, periodic breaks from caffeine (e.g., 1–2 weeks) may be considered as part of a strategy [88,91].

### 7.5. Probiotics

Emerging evidence highlights the critical role of the gut microbiota in regulating systemic inflammation, mood, and overall athletic performance. Probiotic supplementation, which influences the composition and activity of gut microbiota, has garnered attention for its potential benefits in athletic populations. While most studies have focused on endurance sports, recent findings suggest that probiotics may also benefit athletes engaged in high-intensity, anaerobic sports such as weightlifting.

Probiotics, particularly strains of Lactobacillus and Bifidobacterium, have been shown to reduce systemic inflammation by modulating pro-inflammatory cytokines, such as TNF-α and IL-6, and enhancing anti-inflammatory responses [94,95]. This anti-inflammatory effect is particularly relevant for weightlifting athletes, where repetitive high-intensity training can lead to muscle damage, delayed recovery, and chronic low-grade inflammation.

Moreover, the gut–brain axis plays a key role in mood regulation and psychological well-being, which are essential for optimal athletic performance. Probiotics may reduce symptoms of anxiety, depression, and perceived stress by influencing the production of neurotransmitters, such as serotonin and γ-aminobutyric acid (GABA), derived from gut microbiota activity [96]. A recent review by Clark and Mach (2016) highlighted the beneficial effects of probiotics on mood stabilization and cognitive function, suggesting that athletes experiencing high mental stress may benefit from probiotic supplementation as part of a holistic recovery strategy [97].

In addition to their role in inflammation and mood, probiotics may enhance performance indirectly by improving nutrient absorption, immune function, and gastrointestinal (GI) health. GI disturbances, which are commonly reported among athletes consuming high-protein diets, may impair training consistency and performance. Probiotics have been shown to alleviate these issues, improving digestive efficiency and nutrient bioavailability, both of which are critical for weightlifters requiring optimal recovery and muscle adaptation [95].

**Table 2:** Supplementation use strategies.

Supplement	Mechanism of Action	Usage Strategy	Key References
Creatine	Enhances ATP synthesis in muscles, supporting rapid bursts of energy and recovery during high-intensity efforts.	3–5 g/day or 0.3 g/kg body weight. Rapid loading phase: 20 g/day for 5–7 days, followed by 3–5 g/day maintenance.	[2,14,15,74,75,76,77]
BCAAs	Stimulates muscle protein synthesis (mTORC1 pathway), reduces muscle soreness, and accelerates recovery post-exercise.	5–10 g/day before, during, or after exercise. Consuming post-training accelerates muscle repair and reduces fatigue.	[44,47,48,78,79,80,81]
Beta-alanine	Increases carnosine levels to buffer muscle pH, delaying fatigue during high-intensity training.	3–6 g/day for 4–8 weeks. Split doses (e.g., 2 × 2 g/day) to minimize side effects like paraesthesia.	[16,17,82,83,84,85,86]
Caffeine	Stimulates the central nervous system, reduces fatigue perception, and improves focus and exercise efficiency.	3–6 mg/kg body weight 30–60 min pretraining. Periodic breaks (1–2 weeks) recommended to maintain effectiveness.	[83,87,88,89,90,91,92,93]
Probiotics	Modulates gut microbiota to reduce systemic inflammation, improve mood regulation (gut–brain axis), and enhance GI health.	No standardized strategy yet. Further research required for strain specificity, dosage, and anaerobic performance benefits.	[94,95,96,97]

However, while the potential benefits of probiotics are promising, the development of usage strategies or practical guidelines for athletes remains limited due to a lack of evidence, particularly in non-endurance sports such as weightlifting. This limitation underscores the need for further research to establish specific probiotic strains, optimal dosages, and long-term effects on anaerobic performance markers, such as maximal strength, power output, and recovery kinetics. The absence of conclusive data on practical implications should be recognized as a current research limitation and an important area for future investigation.

## 8. Future Research Directions and Issues to Address

The current research has clearly demonstrated the importance of nutritional strategies in improving the performance and training adaptation of weightlifters. Various supplements and appropriate nutrient timing have been shown to play a significant role in strength development and fatigue recovery. However, there are several key limitations in the existing studies that remain critical issues to be addressed in future research.

### 8.1. Lack of Research on Female Weightlifters

Most nutritional research has been conducted on male athletes, whereas studies on female athletes remain relatively scarce. In particular, there is a lack of research analyzing the impact of nutrient intake and training on the hormonal cycles of female weightlifters. McNulty et al. (2020) outlined the relationship between the menstrual cycle and athletic performance in female athletes and reported that appropriate nutrient supplementation during specific phases of the menstrual cycle can help reduce fatigue and injury risk [98,99,100]. However, these studies were conducted on general athletes, and research specifically targeting female weightlifters is limited. Future studies should focus on sex-specific differences in nutritional strategies, with an emphasis on the influence of menstrual cycles on the performance, training, and recovery of female weightlifters.

Hormonal fluctuations across the menstrual cycle significantly affect nutrient metabolism and muscle recovery. During the luteal phase, elevated progesterone levels reduce insulin sensitivity, necessitating tailored carbohydrate intake [101]. Additionally, higher estrogen levels during the follicular phase enhance lipid oxidation, which may influence endurance performance and recovery [102]. These insights underscore the need for phase-specific nutritional strategies to optimize training and recovery.

To address these challenges, personalized training regimens tailored to the menstrual cycle are essential. McNulty et al. (2020) suggested that during the early follicular phase, low-intensity training could promote recovery, while higher-intensity training may be emphasized post-ovulation, when estrogen levels peak [99]. Such adjustments could help mitigate fatigue and strength declines linked to hormonal fluctuations, which are particularly significant for female weightlifters relying on high-intensity strength output.

Future research should investigate the complex relationship between hormonal fluctuations, nutrient metabolism, and muscle recovery in female weightlifters. Comprehensive studies that incorporate key performance determinants are necessary to develop evidence-based strategies, enabling female athletes to overcome hormonal challenges and achieve optimal performance.

### 8.2. Optimization of Personalized Nutrition Strategies

Recent studies have suggested the effectiveness of personalized nutritional strategies based on individual genetic variations, such as single-nucleotide polymorphisms (SNPs) and metabolic characteristics [103,104]. However, a gene–diet interaction (nutrigenomics) may not directly correlate with measurable performance outcomes, such as endurance capacity, speed, or strength. Instead, it may influence intermediate markers, such as body composition or bioactive compounds, which are important for athletic performance, reducing injury risk and supporting recovery after training. Guest et al. (2018) reported that gene-based personalized nutrition strategies have a positive impact on endurance enhancement [105,106]; however, the effects of such strategies on high-intensity sports, such as weightlifting, which relies heavily on explosive strength, remain largely unexplored. Therefore, further research is needed to evaluate how personalized nutritional strategies influence athletic performance and adaptation to training [24]. Additional in-depth studies are required to examine how subtle genetic and physiological differences among individuals affect their long-term athletic development and performance.

### 8.3. Interaction Between Psychological Factors and Nutritional Intake

The relationship between nutrition and psychological stability is a crucial research topic for the future. Psychological stress and fatigue directly affect athletic performance and may interact with nutritional intake [107,108]. Although several studies have evaluated the effects of macronutrients, such as proteins, carbohydrates, and fats, as well as micronutrients such as vitamins, antioxidants, probiotics, and amino acids, on stress responses and recovery processes [107], research specifically targeting weightlifters remains limited. Thus, further studies are required to examine the relationship between nutritional intake and psychological stability, especially when managing pre-competition stress and recovery.

### 8.4. Long-Term Effects of Nutritional Management

Several studies have focused on the short-term effects of nutritional strategies; however, the long-term impact of nutritional management on the entire career of weightlifters remains uncertain. In particular, there is a need to evaluate the long-term effects of extreme diet or supplement use on performance, injury risk, and overall health. This is important for ensuring athletes’ healthy retirement and quality of life after competitive careers.

### 8.5. Safety of Supplements and Doping Concerns

Although supplements, such as creatine, beta-alanine, and caffeine, may be effective in improving performance, concerns regarding their long-term safety still exist [109,110]. Some supplements are classified as banned substances under international doping regulations, posing a serious risk to athletes. Baume et al. (2019) warned that trace amounts of banned substances in supplements could lead to unexpected doping violations in athletes [109,110,111,112]. These issues emphasize the importance of rigorous testing to ensure the safety and purity of supplements used by athletes.

While supplements like branched-chain amino acids (BCAAs) and beta-alanine provide notable performance benefits, chronic overuse may have adverse effects. For instance, excessive BCAA intake has been shown to impair serotonin production, potentially exacerbating central fatigue during prolonged exercise [113]. Similarly, prolonged use of beta-alanine has been associated with paraesthesia, a non-serious but discomforting side effect characterized by tingling sensations [114].

Additionally, restrictive diets that are often adopted in weight-class sports, such as weightlifting, can exacerbate these risks. These diets may lead to nutrient deficiencies and metabolic disturbances, potentially impairing both health and performance. Therefore, balanced dietary approaches under professional supervision are essential to maximize the benefits of supplements while minimizing risks. Future research should focus on evaluating the long-term safety of these supplements and developing strategies to prevent doping violations and adverse health outcomes.

## 9. Conclusions

This study analyzed the importance of nutritional strategies in enhancing the performance of weightlifters. Weightlifting is a high-intensity sport that requires explosive power within a short period, and nutritional management plays a crucial role in performance and recovery. Appropriate intake of carbohydrates, proteins, and fats is essential for energy supply, muscle recovery, and fatigue reduction. Notably, the importance of nutrient timing is also highlighted. Sufficient glycogen storage before training and intake of proteins and carbohydrates after training were found to be critical factors in maximizing muscle resynthesis and fatigue recovery.

Additionally, this study emphasized the effectiveness of supplement-use strategies. Creatine enhances strength and explosive power, caffeine contributes to improved focus and reduced fatigue, and beta-alanine delays fatigue by reducing lactate accumulation. These supplements can significantly enhance performance when administered at the appropriate time and in appropriate amounts.

The importance of personalized nutritional strategies has been highlighted. Recent advances in genetic and metabolomic analyses have made it possible to tailor the nutritional intake to meet the physiological needs of each athlete. This is not only important for improving performance but also for preventing injuries during training and managing fatigue more effectively.

However, there are several key limitations to existing research, and future studies are necessary to address these issues. First, there is a lack of research focusing on female weightlifters, making it essential to conduct studies that clearly analyze the differences in nutritional strategies based on sex. Second, more research is needed to determine the long-term effects of personalized nutritional strategies on actual performance, particularly in high-intensity sports, such as weightlifting, where explosive power is crucial. Third, further research is needed to explore the relationship between nutritional intake and psychological stability. Psychological stress and fatigue directly impact performance, and it is important to understand how nutrition interacts with these factors. Fourth, the safety of the supplements and concerns regarding doping must be further investigated. Although supplements, such as creatine, beta-alanine, and caffeine, can have positive effects on performance, it is necessary to carefully review their long-term safety and potential interactions with doping regulations.

In conclusion, sustainable nutritional management strategies are necessary to enhance weightlifter performance. Rather than prioritizing short-term gains through extreme diets or excessive reliance on supplements, it is crucial to adopt a balanced nutritional approach coupled with systematic management for long-term performance maintenance. The nutritional strategies outlined in this review were derived from a comprehensive synthesis of various research findings, highlighting their potential applicability to weightlifters. However, it is essential to conduct further studies that consider the individual characteristics and physiological differences among athletes. This will enable the refinement and validation of these strategies to support weightlifters in maintaining consistent top-level performance throughout prolonged competitive seasons.

## Figures and Tables

**Table 1:** Practical nutrient timing strategies.

Training Stage	Timing	Recommended Nutrient Ratio	Recommended Food/Supplements	Function
Pre- training	2–3 h before training	- High carbs: 1–4 g/kg - Moderate protein: 0.15–0.25 g/kg - Low fat	Whole-grain bread/pasta, oatmeal, bananas, low-fat yogurt, smoothies	Prepares energy and optimizes glycogen storage
During-training	Every 15–20 min. (>45 min)	- Fast-absorbing carbs: 30–60 g/h - Fluids: 500–1000 mL/h	Sports drinks (6–8% carbs), carbohydrate gels, electrolyte drinks	Maintains energy levels and hydration
Post-training	Within 30 min + meal within 2 h	- Carb–Protein = 3:1 or 4:1 - Carbs: 1.0–1.2 g/kg - Protein: 0.2–0.4 g/kg	Whey protein shakes, bananas, carb drinks, smoothies, cereal with milk	Promotes glycogen replenishment and muscle recovery

## Data Availability

No new data were created or analyzed in this study. Data sharing is not applicable to this article.
